# Gramicidin A: A New Mission for an Old Antibiotic

**DOI:** 10.15586/jkcvhl.2015.21

**Published:** 2015-01-18

**Authors:** Justin M. David, Ayyappan K. Rajasekaran

**Affiliations:** 1 Department of Biological Sciences, University of Delaware, Newark, DE, USA; 2 Therapy Architects, LLC, Wilmington, DE, USA.

## Abstract

Gramicidin A (GA) is a channel-forming ionophore that renders biological membranes permeable to specific cations which disrupts cellular ionic homeostasis. It is a well-known antibiotic, however it’s potential as a therapeutic agent for cancer has not been widely evaluated. In two recently published studies, we showed that GA treatment is toxic to cell lines and tumor xenografts derived from renal cell carcinoma (RCC), a devastating disease that is highly resistant to conventional therapy. GA was found to possess the qualities of both a cytotoxic drug and a targeted angiogenesis inhibitor, and this combination significantly compromised RCC growth in vitro and in vivo. In this review, we summarize our recent research on GA, discuss the possible mechanisms whereby it exerts its anti-tumor effects, and share our perspectives on the future opportunities and challenges to the use of GA as a new anticancer agent.

## Introduction

The plasma membrane physically separates the intracellular components of a cell from the extracellular environment, and its integrity is absolutely essential to sustain cellular functions. Living cells carefully control their intracellular ionic milieu in order to regulate virtually all aspects of cellular biology, including membrane potential, cell volume, cellular pH, solute transport, metabolism, proliferation, survival, and signaling. Disruption of transmembrane ion concentration gradients compromises the ability of cells to properly regulate their internal environment (1), and drugs which have this effect are known as ionophores. These drugs are classified on the basis of their mechanism of action; mobile-carriers complex with metal cations to shield their hydrophilic charge from the hydrophobic interior of the lipid bilayer, and channel-formers insert into the membrane to form hydrophilic pores that permit the rapid passage of select cations through membranes. Many ionophores are produced naturally by various microorganisms in order to defend against competing microbes, and these drugs exhibit broad-spectrum antibiotic properties against Gram-positive bacteria, fungi, parasites, and viruses (2).

Ionophores have traditionally found utility as antibiotics in veterinary medicine and as growth-promoting feed additives for agriculture (1, 2), but research over the past decade has now recognized that they also possess extraordinary anticancer properties. The vast majority of this work has focused on the mobile-carriers monensin and salinomycin. These agents have been shown to induce antiproliferative and cytotoxic effects, overcome therapy resistance, target cancer stem-like cells, and disrupt specific oncogenic signaling pathways in a diverse array of cancer types (reviewed in (2–5)). Furthermore, salinomycin has been used in a small “first-in-man” pilot study with two patients. It was reported to induce tumor/metastasis regression, partial clinical response, and decreased levels of circulating tumor markers without any of the severe and long-term side effects that are commonly observed with conventional chemotherapeutics (4). Continued clinical development of salinomycin is ongoing, and in 2012, the pharmaceutical companies Eisai and Verastem joined together to develop a “proprietary analog of salinomycin” to use as a Wnt inhibitor and anti-cancer stem cell drug for breast cancer.

**Figure 1.**
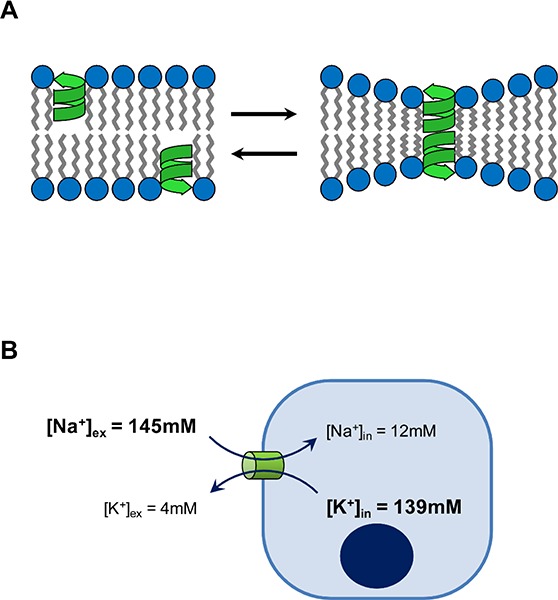
Mechanism of action of gramicidin A. (A) Gramicidin monomers form a β-helix conformation within membranes. Dynamic dimerization of two monomers forms the functional channel, which consequently induces local membrane deformation. (B) Cells maintain a low concentration of intracellular Na+ and a high concentration of intracellular K+ relative to the extracellular environment. Formation of the gramicidin channel (green cylinder) upsets this balance by permitting the passive diffusion of these cations along their respective concentration gradients (arrows) resulting in Na+ influx and K+ efflux.

In contrast to the mobile-carriers, the potential anticancer properties of channel-formers have been largely overlooked. Gramicidin A (GA) is the simplest and best-characterized channel-forming ionophore. It was the very first antibiotic to be isolated and used in a clinical setting, and its initial success paved the way for the clinical development of penicillin and the dawn of the antibiotic era (6). Structurally, GA is a short linear peptide of 15 alternating L- and D- amino acids with a formyl group at the N-terminus and ethanolamine at the C-terminus. GA is extremely hydrophobic, and within biological membranes two GA monomers dimerize end-to-end to form an unusual β-helix nanopore that spans the membrane (7) **(Figure 1A**. Water and inorganic monovalent cations can freely diffuse through the channel formed by GA dimers, and in biological systems this results in Na+ influx/K+ efflux, membrane depolarization, osmotic swelling, and cell lysis (7, 8) **(Figure 1B)**. GA is well known to display potent broad-spectrum antibiotic activity (9–12), and we can now confirm that it also exhibits compelling anticancer properties that are both similar to, and distinct from, the mobile-carrier ionophores.

## Gramicidin A is cytotoxic

In our initial study (13), we evaluated the cytotoxicity of GA using a panel of human cancer cell lines derived from renal cell carcinoma (RCC). RCC is a relatively rare but deadly disease that is histologically heterogeneous and highly resistant to both chemotherapy and radiation. The 5-year disease-specific survival rate for invasive RCC is only 10% (14, 15). We found that treatment with GA decreased the viability of all six of the RCC cell lines tested at submicromolar concentrations (all IC50 < 1.0µM). GA was uniformly toxic regardless of histological subtype or the expression of various molecular markers of relevance to RCC pathophysiology. This finding indicates that GA may be effective in multiple RCC subtypes, which is important because there are as yet no established therapies for the more rare subtypes of RCC (papillary, chromophobe, collecting duct carcinoma, etc.). When we compared GA to the ionophore monensin, a mobile-carrier with similar cation selectivity, we found that GA reduced cell viability equal to or even greater than monensin depending on the cell line tested. However, further examination revealed that whereas monensin provoked apoptotic responses in treated cells, GA induced cell death through a necrotic mechanism that was associated with profound ATP depletion elicited by a blockade of both the oxidative phosphorylation and glycolytic metabolic pathways. GA was also found to effectively suppress tumor growth in vivo.

**Figure 2:**
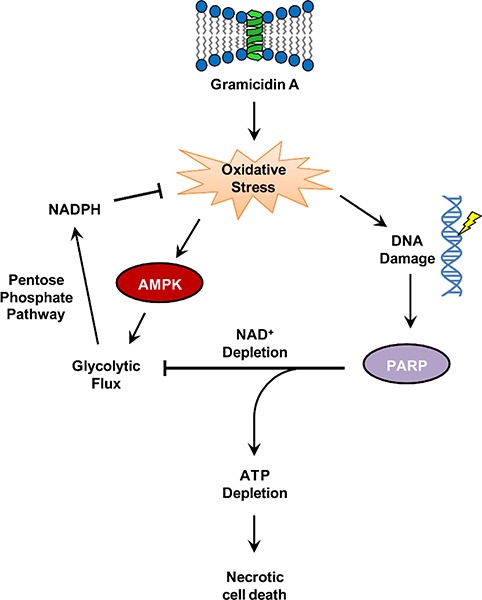
Proposed model of GA cytotoxicity. GA may induce oxidative stress, which can activate AMPK to increase glycolytic flux. This in turn can increase NADPH production via the pentose phosphate pathway, and NADPH regenerates glutathione to detoxify ROS. Oxidative stress also damages DNA leading to the activation of PARP. Overactive PARP depletes NAD+, which inhibits glycolysis leading to ATP depletion and subsequent necrotic cell death.

Collectively, this work demonstrated that perturbation of Na+ and K+ homeostasis by GA impairs cellular metabolism and starves cancer cells of energy. Precisely how this occurs remains to be fully determined, however our evidence supports a model in which oxidative stress is a potential link between GA and energy depletion **(Figure 2)**. Oxidative stress appears to be a common feature of ionophores as both monensin and salinomycin were reported to increase the production of reactive oxygen species (ROS) (16–19). Cells respond to oxidative stress by upregulating ROS detoxifying pathways, and nicotinamide adenine dinucleotide phosphate (NADPH) is a crucial coenzyme that is required for the regeneration of reduced glutathione that is used to detoxify ROS (20). AMP-activated protein kinase (AMPK) was recently shown to increase NADPH production via enhancing glycolytic flux (21), and we observed both increased AMPK activation and a transient initial increase in glycolysis in GA-treated cells. If GA does in fact induce oxidative stress, then it is possible that AMPK responds by upregulating glycolysis to enhance NADPH production in order to alleviate this stress.

In addition, oxidative stress by ionophores damages DNA (16–19). Cells use the enzyme poly (ADP-ribose) polymerase (PARP) to signal damaged DNA by catalyzing the addition of ADP-ribose moieties to nuclear proteins at the site of damage in a reaction that consumes NAD+ (22). In the case of extensive DNA damage, PARP can become overstimulated and deplete cellular NAD+ (22). Glycolysis depends upon the reduction of NAD+ to NADH, and loss of NAD+ blocks glycolysis (22). We did not observe PARP cleavage (inactivation) in GA-treated cells, but we did observe a marked decrease in cellular redox activity and eventual loss of glycolytic activity, suggesting that NAD+ may have been depleted by treatment with GA. Loss of glycolysis would impair NADPH production and rapidly deplete ATP, ultimately leading to necrotic cell death. This mechanism of bioenergetics catastrophe leading to necrosis has been reported for DNA damaging alkylating agents (e.g. nitrogen mustards) (23), suggesting that GA shares important characteristics with conventional chemotherapeutics. Experimental validation of this proposed model **(Figure 2)** would provide key insights into the mechanism of cytoxicity by gramicidin A.

## Gramicidin A inhibits angiogenesis

### Hypoxia and RCC

Oxygen deprivation is a common feature of solid tumors as the tumor micro environment is characterized by a steep oxygen concentration gradient that regularly experiences temporal fluctuations in oxygenation. Accordingly, tumors exhibit many molecular and biochemical features associated with the cellular response to low oxygen (hypoxia), which is controlled by the transcription factor hypoxia-inducible factor (HIF). Numerous functional investigations have revealed that HIF promotes tumor growth, vascularization, and metastatic spread, and a large body of clinical evidence has linked HIF activation with cancer progression, and reduced patient survival (24–26). RCC tumors in particular display features associated with chronic hypoxia responses (27), and it is now recognized that constitutive activation of HIF is a key etiologic feature of RCC.

HIF exists as a heterodimer that consists of an oxygen-sensitive α-subunit and a constitutively expressed β-subunit (HIF-β) (28). Regulation of HIF activity is mediated by strict control of the protein levels of the α-subunits. When oxygen levels are adequate (normoxia), HIF-α is rapidly hydroxylated and bound by the von Hippel-Lindau tumor suppressor protein (VHL) which promotes the ubiquitylation and subsequent degradation of HIF-α (27). Conversely, HIF-α stabilizes in hypoxic conditions as O2 deprivation inhibits protein hydroxylation. Constitutive activation of HIF occurs in RCC through the loss of VHL expression/activity in the clear cell subtype (ccRCC), and through additional VHL-independent means in other subtypes (14). Anti-angiogenesis therapies that antagonize HIF function (e.g. sunitinib, sorafenib, bevacizumab, etc.) have succeeded in increasing progression-free survival and quality of life for ccRCC patients, however durable and complete remissions remain rare (29).

### Gramicidin A Inhibits HIF

Studies conducted over the past 10–15 years have demonstrated that a diverse array of chemotherapeutic agents, including topoisomerase inhibitors, microtubule-targeting drugs, and anthracyclines, can inhibit HIF transcriptional activity (24). Furthermore, low-dose cyclophosphamide given at more frequent intervals has been shown to block tumor angiogenesis (30). Given the aforementioned cytotoxic similarities between GA and chemotherapy drugs, we sought to examine the effects of GA upon hypoxia responses in RCC cells. We discovered that treatment of cells with GA reduced the expression of the HIF-1α and HIF-2α isoforms in both normoxic and hypoxic conditions. This in turn suppressed HIF-dependent hypoxia responses and occurred even at doses lower than those used in our prior cytotoxicity studies. Comparison of GA with the mobile-carriers monensin, valinomycin, and calcimycin showed that only GA elicited a dramatic and persistent decrease in HIF-1α and HIF-2α expression. These effects only occurred in VHL-positive but not VHL-negative cell lines, and mechanistic examination revealed that GA specifically upregulates the VHL tumor suppressor to accelerate the O2-dependent destabilization of HIF. These effects were confirmed in vivo, as treatment with GA reduced the growth and angiogenesis in VHL-positive RCC tumors.

## VHL upregulation by Gramicidin A

The anti-angiogenic effects of GA raise several provocative questions and possibilities. First, precisely how perturbing the intracellular ionic milieu affects VHL expression is not fully understood. GA exhibits similar sensitivity for Na+ and K+ (31) and induces the simultaneous influx of Na+ and efflux of K+ in living cells. When we compared GA with three mobile-carrier ionophores, only valinomycin provoked a partial decrease in HIF expression. Since valinomycin is highly selective for K+ over Na+ (32), this result suggests that increased VHL expression is due primarily to the loss of intracellular K+, assuming the mechanism of HIF downregulation is identical for both drugs. Further experiments will be necessary to confirm this supposition. Second, our results showed that only VHL protein increased in GA-treated cells implying that either the translation of VHL transcripts or the stability of VHL protein was increased. Factor(s) that regulate VHL mRNA translation have yet to be identified, but several factors are known to influence VHL protein stability. VHL is stabilized when bound to its associated ubiquitin ligase components (elongins B and C, RBX1, cullin 2) (33), and GA may promote this binding. Alternatively, several proteins are known to specifically target and destabilize VHL: 1) E2-EPF ubiquitin carrier protein is another ubiquitin ligase component that directly targets VHL for proteasomal degradation and is expressed in primary and metastatic tumors (34); 2) casein kinase 2 destabilizes VHL through phosphorylation of serines 33, 38, and 43 and is upregulated in most human cancers (35); 3) transglutaminase 2 is a crosslinking enzyme that causes VHL degradation by polymerization and is also overexpressed in many cancers (36). Whether GA inhibits any of these cancer-associated proteins to stabilize VHL expression remains to be determined. Third, our findings indicate that upregulation of VHL by GA blocks tumor angiogenesis and growth, yet we found no relationship between VHL expression and in vitro viability in response to GA (13). This finding was actually not surprising as studies have reported that VHL overexpression in naturally VHL-deficient cell lines caused dramatic suppression of in vivo tumor formation and growth without concomitant inhibition of in vitro cell growth (37, 38). However, exactly how much of the reduction in tumor growth by GA is due to direct cytotoxicity (VHL-independent) as opposed to the blockade of tumor angiogenesis (VHL-dependent) is not yet known.

Lastly, it has become increasingly apparent in recent years that VHL suppresses tumorigenesis not only through the downregulation of HIF, but also through a myriad of HIF-independent mechanisms. VHL has been shown to directly bind both fibronectin and collagen IV alpha 2 and promote the proper assembly of the extracellular matrix, and loss of VHL disrupts the normal tissue and extracellular matrix architecture in a way that better facilitates tumor growth, invasion, and blood vessel infiltration (39). VHL also downregulates integrins which prevent cell motility and invasion by preserving the cell-cell adhesions of both the tight and adherens junctions (39). Furthermore, VHL stabilizes microtubules at the cell periphery, which positively regulates the biogenesis and function of the primary cilium. The primary cilium is a microtuble-based organelle found in all cells that functions as a chemo-, osmo-, and mechano-sensor of the extracellular environment, and its loss in VHL-deficient kidney cells leads to inappropriate proliferation and the formation of preneoplastic renal cysts (i.e. polycystic kidney disease) (39, 40). Finally, VHL stabilizes the fellow tumors suppressor proteins p53 and Jade-1 (gene for apoptosis and differentiation in epithelia), which preserves DNA damage responses and inhibits oncogenic Wnt/β-catenin signaling, respectively (40, 41). An exciting proposition to consider is whether upregulation of VHL by GA promotes these additional HIF-independent mechanisms to block tumor growth and development. Validation of this notion would broaden the therapeutic appeal of GA as a treatment for VHL-positive cancers of the kidney and other tissues alike.

## Future development of GA

Generalized toxicity is a significant challenge to the development of ionophores as therapies for human cancer. GA causes hemolysis and is toxic to the liver, kidney, meninges, and olfactory apparatus (7, 42), and polyether mobile-carrier ionophores are also toxic and elicit neurological side effects (1, 43). However, a variety of normal and nonmalignant cells were reported to be less sensitive to mobile-carrier ionophores (16–18, 44, 45) and murine xenograft experiments from ours and other investigators have demonstrated the in vivo efficacy of ionophores without significant side effects (46–50). Furthermore, salinomycin was shown to be effective in two human cancer patients without eliciting any severe toxicities (4). Nevertheless, a comprehensive understanding of effects of ionophore drugs upon cancer cells vs. normal tissues is currently lacking and will be necessary before clinical development can progress to a larger scale.

The generalized toxicity of GA can be alleviated by intratumoral injection. This method of administration improves the therapeutic index of drugs by concentrating the drug at the tumor site only to spare the rest of the body. We found intratumoral injection of GA to be both safe and effective in our murine xenograft studies. Through the use of X-ray computed tomography, intratumoral injection in the clinic is now possible for metastatic and/or inoperable tumors, and we suggest that wider use of the technique will allow agents such as GA to advance into clinical use more rapidly.

Chemical modification or mutation of the GA peptide has proven effective at increasing microbial targeting and decreasing non-specific toxicity (7, 8, 51, 52). Such mutagenesis approach could be utilized to identify a non-toxic but efficacious form of GA that could be used systemic delivery for treating tumors in in vivo. Alternatively, encapsulation of GA in nanoparticles targeted to the tumor could be used to safely deliver GA for treatment purposes. A recent report by Wijesinghe et al. used a novel pH-sensitive liposomal approach to deliver encapsulated GA into the membranes of cancer cells, resulting in cancer cell death (53). Such an approach could be used to target cancer cells within the acidic tumor microenvironment only, thereby reducing non-specific toxicity by sparing normal tissues.

## Conclusion

GA, the channel-forming ionophore, has cytotoxic and antiangiogenic activities in RCC tumors. The cytotoxic activity is due to ATP depletion, and the anti-angiogenic effect is due to the inhibition of HIF via the induction of endogenously expressed VHL. Our in vitro and in vivo studies strongly suggest that GA has the potential to be developed into a therapeutic agent for RCC and possibly other cancers.

## References

[1] Kart A, Bilgili A. (2008). Ionophore Antibiotics: Toxicity, Mode of Action and Neurotoxic Aspect of Carboxylic Ionophores.. Journal of Animal and Veterinary Advances.

[2] Kevin DA, Meujo DAF, Hamann MT. (2009). Polyether ionophores: broad-spectrum and promising biologically active molecules for the control of drug-resistant bacteria and parasites.. Expert Opinion on Drug Discovery.

[3] Huczynski A. (2012). Polyether ionophores-promising bioactive molecules for cancer therapy.. Bioorg Med Chem Lett.

[4] Naujokat C, Steinhart R. (2012). Salinomycin as a drug for targeting human cancer stem cells.. J Biomed Biotechnol.

[5] Zhou S, Wang F, Wong ET, Fonkem E, Hsieh TC, Wu JM, Wu E. (2013). Salinomycin: a novel anti-cancer agent with known anti-coccidial activities.. Current medicinal chemistry.

[6] Kelkar DA, Chattopadhyay A. (2007). The gramicidin ion channel: a model membrane protein.. Biochim Biophys Acta.

[7] Wang F, Qin L, Pace CJ, Wong P, Malonis R, Gao J. (2012). Solubilized gramicidin A as potential systemic antibiotics.. Chembiochem.

[8] Otten-Kuipers MA, Beumer TL, Kronenburg NA, Roelofsen B, Op den Kamp JA. (1996). Effects of gramicidin and tryptophan-N-formylated gramicidin on the sodium and potassium content of human erythrocytes.. Mol Membr Biol.

[9] Dubos RJ. (1939). Studies on a Bactericidal Agent Extracted from a Soil Bacillus: I. Preparation of the Agent. Its Activity in Vitro.. J Exp Med.

[10] Dubos RJ, Hotchkiss RD. (1941). The Production of Bactericidal Substances by Aerobic Sporulating Bacilli.. J Exp Med.

[11] Bourinbaiar AS, Coleman CF. (1997). The effect of gramicidin, a topical contraceptive and antimicrobial agent with anti-HIV activity, against herpes simplex viruses type 1 and 2 in vitro.. Arch Virol.

[12] Moll GN, van den Eertwegh V, Tournois H, Roelofsen B, Op den Kamp JA, van Deenen LL. (1991). Growth inhibition of Plasmodium falciparum in in vitro cultures by selective action of tryptophan-N-formylated gramicidin incorporated in lipid vesicles.. Biochim Biophys Acta.

[13] David JM, Owens TA, Barwe SP, Rajasekaran AK. (2013). Gramicidin A induces metabolic dysfunction and energy depletion leading to cell death in renal cell carcinoma cells.. Mol Cancer Ther.

[14] Baldewijns MM, van Vlodrop IJ, Schouten LJ, Soetekouw PM, de Bruine AP, van Engeland M. (2008). Genetics and epigenetics of renal cell cancer.. Biochim Biophys Acta.

[15] Patard JJ (2005). Prognostic value of histologic subtypes in renal cell carcinoma: a multicenter experience.. J Clin Oncol.

[16] Ketola K, Vainio P, Fey V, Kallioniemi O, Iljin K. (2010). Monensin is a potent inducer of oxidative stress and inhibitor of androgen signaling leading to apoptosis in prostate cancer cells.. Mol Cancer Ther.

[17] Ketola K, Hilvo M, Hyotylainen T, Vuoristo A, Ruskeepaa AL, Oresic M, Kallioniemi O, Iljin K. (2012). Salinomycin inhibits prostate cancer growth and migration via induction of oxidative stress.. Br J Cancer.

[18] Kim KY, Yu SN, Lee SY, Chun SS, Choi YL, Park YM, Song CS, Chatterjee B, Ahn SC. (2011). Salinomycin-induced apoptosis of human prostate cancer cells due to accumulated reactive oxygen species and mitochondrial membrane depolarization.. Biochem Biophys Res Commun.

[19] Verdoodt B, Vogt M, Schmitz I, Liffers ST, Tannapfel A, Mirmohammadsadegh A. (2012). Salinomycin induces autophagy in colon and breast cancer cells with concomitant generation of reactive oxygen species.. PLoS One.

[20] Jeon SM, Chandel NS, Hay N. (2012). AMPK regulates NADPH homeostasis to promote tumour cell survival during energy stress.. Nature.

[21] Wu SB, Wei YH. (2012). AMPK-mediated increase of glycolysis as an adaptive response to oxidative stress in human cells: implication of the cell survival in mitochondrial diseases.. Biochim Biophys Acta.

[22] Ying W, Alano CC, Garnier P, Swanson RA. (2005). NAD+ as a metabolic link between DNA damage and cell death.. J Neurosci Res.

[23] Zong WX, Ditsworth D, Bauer DE, Wang ZQ, Thompson CB. (2004). Alkylating DNA damage stimulates a regulated form of necrotic cell death.. Genes Dev.

[24] Semenza GL. (2010). Defining the role of hypoxia-inducible factor 1 in cancer biology and therapeutics.. Oncogene.

[25] Zhong H, De Marzo AM, Laughner E, Lim M, Hilton DA, Zagzag D, Buechler P, Isaacs WB, Semenza GL, Simons JW. (1999). Overexpression of hypoxia-inducible factor 1alpha in common human cancers and their metastases.. Cancer Res.

[26] Talks KL, Turley H, Gatter KC, Maxwell PH, Pugh CW, Ratcliffe PJ, Harris AL. (2000). The expression and distribution of the hypoxia-inducible factors HIF-1alpha and HIF-2alpha in normal human tissues, cancers, and tumor-associated macrophages.. Am J Pathol.

[27] Haase VH. (2012). Renal cancer: Oxygen meets metabolism.. Exp Cell Res.

[28] Wang GL, Jiang BH, Rue EA, Semenza GL. (1995). Hypoxia-inducible factor 1 is a basic-helix-loop-helix-PAS heterodimer regulated by cellular O2 tension.. Proc Natl Acad Sci U S A.

[29] Kirchner H, Strumberg D, Bahl A, Overkamp F. (2010). Patient-based strategy for systemic treatment of metastatic renal cell carcinoma.. Expert Rev Anticancer Ther.

[30] Hahnfeldt P, Folkman J, Hlatky L. (2003). Minimizing long-term tumor burden: the logic for metronomic chemotherapeutic dosing and its antiangiogenic basis.. Journal of theoretical biology.

[31] Christensen HN. (1975). Biological Transport.

[32] Rose M.C. (1974). HRW. Stability of sodium and potassium complexes of valinomycin.. Biochimica et Biophysica Acta.

[33] Kamura T, Brower CS, Conaway RC, Conaway JW. (2002). A molecular basis for stabilization of the von Hippel-Lindau (VHL) tumor suppressor protein by components of the VHL ubiquitin ligase.. J Biol Chem.

[34] oJung CR Hwang KS Yoo J Cho WK Kim JM Kim WH Im DS. E2-EPF UCP targets pVHL for degradation and associates with tumor growth and metastasis. Nat Med 2006 12 809 16 Doi: http://dx.doi.org/10.1038/nm1440 16819549 10.1038/nm1440

[35] Ampofo E, Kietzmann T, Zimmer A, Jakupovic M, Montenarh M, Gotz C. (2010). Phosphorylation of the von Hippel-Lindau protein (VHL) by protein kinase CK2 reduces its protein stability and affects p53 and HIF-1alpha mediated transcription.. Int J Biochem Cell Biol.

[36] Kim DS (2011). Cancer cells promote survival through depletion of the von Hippel-Lindau tumor suppressor by protein crosslinking.. Oncogene.

[37] Gnarra JR, Tory K, Weng Y, Schmidt L, Wei MH, Li H, Latif F, Liu S, Chen F, Duh FM. (1994). Mutations of the VHL tumour suppressor gene in renal carcinoma.. Nat Genet.

[38] Schoenfeld A, Davidowitz EJ, Burk RD. (1998). A second major native von Hippel-Lindau gene product, initiated from an internal translation start site, functions as a tumor suppressor.. Proc Natl Acad Sci U S A.

[39] Jonasch E (2012). State-of-the-science: An update on renal cell carcinoma.. Mol Cancer Res.

[40] Nyhan MJ, O’Sullivan GC, McKenna SL. (2008). Role of the VHL (von Hippel-Lindau) gene in renal cancer: a multifunctional tumour suppressor.. Biochem Soc Trans.

[41] Chitalia VC (2008). Jade-1 inhibits Wnt signalling by ubiquitylating beta-catenin and mediates Wnt pathway inhibition by pVHL.. Nat Cell Biol.

[42] Grayson ML. (2010). Kucers’ the use of antibiotics: a clinical review of antibacterial, antifungal, antiparasitic and antiviral drugs.

[43] Boehmerle W, Endres M. (2011). Salinomycin induces calpain and cytochrome c-mediated neuronal cell death.. Cell Death Dis.

[44] Fuchs D, Heinold A, Opelz G, Daniel V, Naujokat C. (2009). Salinomycin induces apoptosis and overcomes apoptosis resistance in human cancer cells.. Biochem Biophys Res Commun.

[45] Lu D, Choi MY, Yu J, Castro JE, Kipps TJ, Carson DA. (2011). Salinomycin inhibits Wnt signaling and selectively induces apoptosis in chronic lymphocytic leukemia cells.. Proc Natl Acad Sci U S A.

[46] Park WH, Lee MS, Park K, Kim ES, Kim BK, Lee YY. (2002). Monensin-mediated growth inhibition in acute myelogenous leukemia cells via cell cycle arrest and apoptosis.. Int J Cancer.

[47] Gupta PB, Onder TT, Jiang G, Tao K, Kuperwasser C, Weinberg RA, Lander ES. (2009). Identification of selective inhibitors of cancer stem cells by high-throughput screening.. Cell.

[48] Basu D. (2011). Detecting and targeting mesenchymal-like subpopulations within squamous cell carcinomas.. Cell Cycle.

[49] Tang QL. (2011). Salinomycin inhibits osteosarcoma by targeting its tumor stem cells.. Cancer Lett.

[50] Zhang B, Wang X, Cai F, Chen W, Loesch U, Bitzer J, Zhong XY. (2012). Effects of salinomycin on human ovarian cancer cell line OV2008 are associated with modulating p38 MAPK.. Tumour Biol.

[51] Sorochkina AI, Plotnikov EY, Rokitskaya TI, Kovalchuk SI, Kotova EA, Sychev SV, Zorov DB, Antonenko YN. (2012). N-terminally glutamate-substituted analogue of gramicidin A as protonophore and selective mitochondrial uncoupler.. PLoS One.

[52] Lewis JC, Dimick KP, Feustel IC, Fevold HL, Olcott HS (1945). Fraenkel-Conrat H. Modification of Gramicidin through Reaction with Formaldehyde.. Science.

[53] Wijesinghe D, Arachchige MC, Lu A, Reshetnyak YK, Andreev OA. (2013). pH dependent transfer of nano-pores into membrane of cancer cells to induce apoptosis.. Scientific reports.

